# LINC00924-induced fatty acid metabolic reprogramming facilitates gastric cancer peritoneal metastasis via hnRNPC-regulated alternative splicing of Mnk2

**DOI:** 10.1038/s41419-022-05436-x

**Published:** 2022-11-23

**Authors:** Qiuming He, Chaogang Yang, Zhenxian Xiang, Guoquan Huang, Haitao Wu, Tingna Chen, Rongzhang Dou, Jialing Song, Lei Han, TianTian Song, Shuyi Wang, Bin Xiong

**Affiliations:** 1 Department of Gastrointestinal Surgery, Zhongnan Hospital of Wuhan University, Wuhan, 430071 China; 2 Department of Gastric and Colorectal Surgical Oncology, Zhongnan Hospital of Wuhan University, Wuhan, 430071 China; 3 Hubei Key Laboratory of Tumor Biological Behaviors, Wuhan, 430071 China; 4 Hubei Cancer Clinical Study Center, Wuhan, 430071 China

**Keywords:** Cancer metabolism, Metastasis

## Abstract

The molecular mechanism underlying gastric cancer (GC) peritoneal metastasis (PM) remains unclear. Here, we identified LINC00924 as a GC PM-related lncRNA through Microarray sequencing. LINC00924 was highly expressed in GC, and its high expression is associated with a broad range of PM. Via RNA sequencing, RNA pulldown assay, mass spectrometry, Seahorse, Lipidomics, spheroid formation and cell viability assays, we found that LINC00924 promoted fatty acid (FA) oxidation (FAO) and FA uptake, which was essential for matrix-detached GC cell survival and spheroid formation. Regarding the mechanism, LINC00924 regulated the alternative splicing (AS) of Mnk2 pre-mRNA by binding to hnRNPC. Specifically, LINC00924 enhanced the binding of hnRNPC to Mnk2 pre-mRNA at e14a, thus downregulating Mnk2a splicing and regulating the p38 MAPK/PPARα signaling pathway. Collectively, our results demonstrate that LINC00924 plays a role in promoting GC PM and could serve as a drug target.

## Introduction

Gastric cancer (GC) is the fourth leading cause of cancer-related deaths worldwide [1]. Peritoneal metastasis (PM) is a frequent cause of mortality in GC patients [2]. Despite the common occurrence of GC PM, our current understanding of the mechanism is unclear, which limits our ability to optimize treatment and improve survival. Metastasis is a highly inefficient process because cancer cells need to overcome multiple environmental hurdles, such as immune responses [3], oxidative stress [4], and energetic stress [5]. Because of these environmental hurdles, most cancer cells perish, with only a small proportion surviving and growing into metastatic foci [6, 7]. Understanding the molecular mechanisms by which metastasizing cancer cells overcome these hurdles can potentially aid the prevention and treatment of GC PM.

Metabolic reprogramming is a characteristic phenotype of cancers that supports specific demands for energy, biosynthesis, and redox maintenance [8]. Recent and, increasing evidence suggests that metastatic cells require metabolic reprogramming to survive and grow in the new environment; this reprogramming, includes changes to pyruvate, lactate, glutamine, and fatty acid (FA) metabolism [9]. Among them, FA metabolic reprogramming, which classically occurs as increased FA oxidation (FAO) and lipid synthesis, may provide cancer cells with a selective advantage in the metastatic process [10, 11]. However, the regulatory mechanisms by which GC cells reprogram FA metabolism during metastasis are unclear.

Long noncoding RNAs (lncRNAs, noncoding RNAs longer than 200 nucleotides) are a class of transcripts without protein-coding potential that have been reported to play a role in FA metabolic reprogramming. LncRNAs may regulate the expression of genes involved in FAO by altering RNA stability [12] and may activate important signaling pathways related to lipid metabolism through a ceRNA (competing endogenous RNA) mechanism [13]. Moreover, a growing number of studies have implicated lncRNAs in alternative splicing (AS) regulatory processes [14–17]. AS is a fundamental mechanism that allows the production of multiple isoforms through the differential processing of introns and exons from a single gene, thereby increasing molecular diversity [18]. LncRNAs can regulate AS by binding to splicing factors associated with pre-mRNA and impinging on chromatin remodeling [14]. However, few studies have investigated the role of lincRNAs in regulating AS in lipid metabolic reprogramming, especially in GC PM.

In this study, we examined lncRNA expression profiles in GC primary foci and peritoneal foci and identified LINC00924 as a GC PM-related lncRNA. We found that LINC00924 was highly expressed in GC patients, and its high expression mediated a broad range of PMs. We demonstrated that LINC00924 regulated GC cell lipid metabolic reprogramming, which subsequently promoted matrix-detached GC cell survival and spheroid formation. Regarding the mechanism, LINC00924 regulated MNK2 pre-mRNA AS by binding to hnRNPC. Specifically, LINC00924 promoted hnRNPC binding to MNK2 pre-mRNA at e14a, thus downregulating Mnk2a splicing and regulating the p38 MAPK/PPARα signaling pathway. Furthermore, an in vivo study revealed that LINC00924 knockdown strongly inhibited the growth of GC and attenuated peritoneal dissemination in vivo. Our results extend the understanding of lncRNA-mediated cancer metastasis, which may be potentially efficacious for the prevention and treatment of GC PM.

## Materials and methods

### Patients and samples

Sixty pairs of GC and ANT samples, 3 paired of ANT, primary GC and PM from GC samples were obtained from the specimen bank of the Cancer Institute of Zhongnan Hospital of Wuhan University. No patient had received radiotherapy or chemotherapy before surgery. All patients provided informed consent for the procedures, and this study was approved by the Ethics Review Board of Zhongnan Hospital of Wuhan University.

### Cell lines and cell culture

Human GC cell lines (MGC803, MKN45, BGC823, AGS, HGC27, SGC7901) and a human stomach epithelial cell line (GES-1) were obtained from the American Type Culture Collection (ATCC, USA). Cells were cultured in RPMI 1640 medium (Gibco, USA) with 10% FBS (Gibco, USA) at 37 °C with 5% CO2.

### Transient transfection and stable transfection

All siRNA duplexes were designed and generated by Gene pharma (Shanghai, China). The sequences of siRNA duplexes were shown in Supplementary Table 1. Transient transfection was performed using Lipo2000 reagent (Invitrogen). LINC00924 overexpression, LINC00924 knockdown and hnRNPC knockdown lentiviral vectors constructed in the Gene pharma (Shanghai, China). Lentiviral infections were done according to the instruction of manufacture. Briefly, virus and polybrene (final 5 μg/ml, Sigma Aldrich, Cat#107689) was added to 25% confluent cells. Fresh media was added 16 h after infection. Media was changed with media containing appropriate antibiotics 48 h after infection. After Puro selection, cells were maintained for at least one day without drug for further experiments.

### Real-time PCR and real-time quantitative PCR

Paired tumor and nontumorous specimens from the primary cohort were subjected to RNA isolation and real-time quantitative PCR (RT–qPCR) using GAPDH as an internal control. Briefly, total RNA was extracted from tissues and cells using TRIzol reagent. RNA was reverse transcribed into cDNA with Prime-Script RT Master Mix (TaKaRa, Kyoto, Japan). qPCR was conducted with SYBR Premix Ex Taq II kits (TaKaRa, Kyoto, Japan). Real-time PCR (RT–PCR) was performed using a one-step RT–PCR kit (QIAGEN) according to the manufacturer’s protocol. The primers used for RT–PCR and RT–qPCR is listed in Supplemental Table 4.

### Western blot assay

Samples for protein analysis were extracted on ice using RIPA lysis buffer. After centrifugation, supernatants were quantified using bicinchoninic acid (BCA) kit. Then, samples were boiled in SDS sample buffer and electrophoresed in 10% SDS–PAGE. After transblotted onto PVDF membranes, blots were incubated with primary antibodies overnight at 4 °C and then incubated with secondary antibodies for 1 h at room temperature. Antibodies used were shown in Supplementary Table 3.

### Colony formation and CCK8 assay

For the colony formation detection, AGS and MGC803 were placed in 6-well plates at a density of 1000 cells per well. After two week’s culture in incubator, the cells were fixed with 4% paraformaldehyde and stained with 0.5% crystal violet. CCK8 assays were performed according to the manufacturer’s manual (Beyotime, China).

### Wound healing assay

GC cells were seeded in 6-well plates and grown until ~80% confluence. A sterile pipette tip was used to make the scratch line. The photos were taken per 12 h and the migration rate was calculated using ImageJ software.

### Transwell migration and invasion assay

Transwell chambers (8 μm pore size; Corning, USA) were used for the migration assay. Cell invasion assays were performed with Matrigel-coated Transwell chambers. MGC803 cells and AGS cells were seeded in the upper chamber and incubated for 48 h. Then, the migrated and invaded cells were fixed in 4% paraformaldehyde and stained with 0.5% crystal violet. Five random fields from each well were counted under a microscope (magnification, ×200).

### Fluorescence in situ hybridization and RNA nuclear-cytoplasmic separation

Fluorescence in situ hybridization (FISH) assays were performed using a FISH kit (GenePharma, Shanghai, China). Briefly, GC cells were fixed in 4% formaldehyde for 20 min and washed with PBS. The cells were then incubated with a FISH probe in hybridization buffer. DAPI was used to counterstain the nuclei, and images were obtained with microscopy. Nuclear and cytosolic fractionation assays were performed using a nuclear and cytoplasmic extraction kit according to the manufacturer’s protocol.

### RNA pulldown and mass spectrometry

RNA pulldown assays were performed according to the protocol of the RNA pulldown assay kit (BersinBio, Guangzhou, China). Biotin-labeled LINC00924 and LacZ probes were synthesized by GenePharma. The proteins were resolved by SDS–PAGE followed by silver staining. The specific bands were identified by mass spectrometry or western blot (WB).

### RNA sequencing and pathway enrichment analysis

Total RNA was extracted from the NC and LINC00924-OE groups of MGC803 cells using TRIzol reagent (Invitrogen). Quality control, library construction, RNA sequencing, and bioinformatic analysis were performed at BGI (Beijing Genomics Institute). Gene set enrichment analysis (GSEA) of differentially expressed genes was performed on the Dr. Tom network platform at BGI (http://report.bgi.com).

### RNA binding protein immunoprecipitation

The RNA binding protein immunoprecipitation (RIP) assay was performed by using a RIP kit (Millibo, Massachusetts, USA). Briefly, GC cells were lysed with RIP lysis buffer and then incubated with anti-hnRNPC or anti-IgG antibodies. The incubated samples were washed 6 times with RIP wash buffer. Finally, the RNA was extracted, reverse transcribed and measured by qRT–PCR.

### RNA antisense purification

RNA antisense purification (RAP) was performed according to the protocol of the RAP assay kit (BersinBio, Guangzhou, China). After DNA removal, the lysates were co-incubated with Biotin-labeled LINC00924 and LacZ probe (GenePharma Shanghai, China) at 37 °C for 180 min. Probes were subsequently captured by streptavidin-coated magnetic beads. After washing, the eluted RNA was extracted, reverse transcribed and measured by qRT-PCR.

### FAO assay

The FAO rate was measured by using an XFe24 analyzer (Seahorse Bioscience) according to the manufacturer’s instructions. Cells were plated in 24-well XFe24 cell culture plates (20000 cells per well). Then, the cells were incubated in base growth media (Seahorse Bioscience) for 24 h under substrate limitation conditions. Cells were incubated with FAO assay medium for 1 h in a non-CO2 incubator. Etomoxir (20 μM) was added to inhibit FAO. Finally, the plate was placed into an XFe24 analyzer, and the XF Cell Mito Stress Test was run according to the manufacturer’s recommended protocol.

### Lipid droplet staining

Intracellular lipid droplets were stained using BODIPY. After being fixed with 4% paraformaldehyde for 15 min and permeabilization with 0.2% Triton X-100 for 10 min, the cells were incubated with BODIPY (1 μg/ml) overnight at 4 °C. Then, DAPI (0.5 μg/ml) was used for nuclear staining. Finally, the images were visualized by fluorescence microscopy.

### Spheroid formation and cell viability

MGC-803 and AGS cells (3000 cells/well) were seeded in ultralow attachment 6-well plates (Corning, USA). After 5 days, the spheroids were imaged using an Olympus BX5 fluorescence microscope (Olympus Optical, Japan). Then, the cells were collected, and cell viability was assessed using a Calcein-AM/PI Double Stain Kit (Yeasen, Shanghai, China). The cells were observed by fluorescence microscopy, and live cells were stained green, while dead cells were stained red. Spheroid formation and cell viability were then manually examined.

### In vivo xenograft assay

Six-week-old female BALB/c-nu mice were purchased from GemPharmatech (Jiangsu, China). In the subcutaneous tumorigenesis model, 5.0 × 10^6^ cells were injected into the right armpit of the mice subcutaneously. For the tumor peritoneal dissemination assay, 5 × 10^6^ cells in 40 μl of serum-free medium containing 50% Matrigel, were subserosal injected into the greater curvature of the stomach. After 35 days, the animals were euthanized, followed by tumor excision and weight determination. Our animal studies were approved by the Wuhan University Ethics Committee.

### Statistical analysis

The experiments were repeated three times in triplicate, and the results are shown as the mean ± SD. Two-group comparisons were made using two-tailed Student’s *t* tests or chi-square tests. Correlations between continuous variables were analyzed using Spearman correlations. Survival was analyzed by the Kaplan–Meier survival curves. Values of *P* < 0.05 were considered statistically significant.

## Results

### Identification of GC PM-related LINC00924

**Fig. 1:**
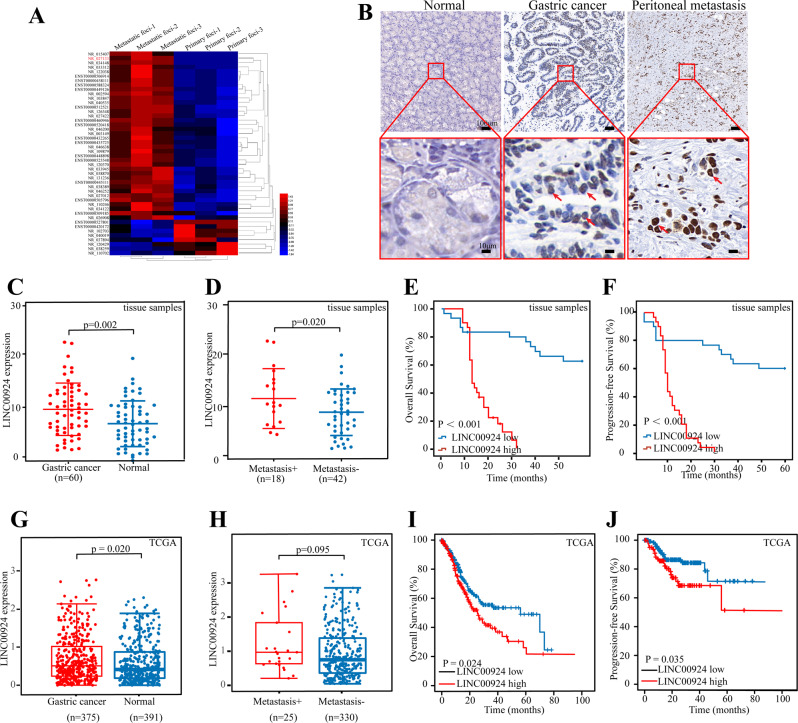
Identification of GC PM related LINC00924. **A** Differential expression of lncRNAs in 3 pairs of GC primary and peritoneal metastatic lesions identified by lncRNA microarrays. **B** Representative ISH staining for LINC00924 expression in normal tissue, GC primary lesions and peritoneal metastatic lesions. **C** qRT–PCR analysis of LINC00924 expression levels in GC and normal tissues. **D** qRT–PCR analysis of LINC00924 expression levels in metastasis-positive (metastasis + ) and metastasis-negative (metastasis -) GC patients. E-F: The associations of LINC00924 expression with five-year OS (**E**) and five-year DFS (**F**) were analyzed by Kaplan–Meier survival analysis. **G**: qRT–PCR analysis of LINC00924 expression levels in GC and normal tissues from TCGA. **H**: qRT–PCR analysis of LINC00924 expression levels in metastasis-positive (metastasis + ) and metastasis-negative (metastasis -) GC patients from TCGA. **I**, **J**: Kaplan–Meier survival analysis curve calculated from 375 GC patients from TCGA. *p < 0.05, **p < 0.01, ***p < 0.001.

In our previous study [19], we performed a microarray assay of peritoneal metastasis lesions and primary gastric cancer foci from three patients without any treatment who were diagnosed with gastric cancer peritoneal metastasis, 38 upregulated and 9 downregulated lncRNAs with fold change >2 and known structural and functional studies were identified (Fig. 1A). Among them, LINC00924 had a significant increase in expression after in vivo environment simulation of peritoneal metastasis and its expression was closely associated with low overall survival [19]. Furthermore, LINC00924 was the second most highly expressed lncRNA in peritoneal metastasis lesions compared to primary gastric cancer foci. Thus, LINC00924 was selected as the research object and we next sought to investigate the potential role of LINC00924 in GC PM. First, we performed RNA-ISH experiments to determine whether LINC00924 expression could be directly visualized in tissue specimens. The results demonstrated that LINC00924 was specifically localized within the nucleus and was highly expressed in PM compared with GC and normal tissues. Representative images are shown in Fig. 1B. Next, quantification of LINC00924 in clinical tissue samples was performed by RT–qPCR, which revealed that LINC00924 levels were significantly higher in GC than in normal tissues (Fig. 1C) and in metastasis-positive GC than in metastasis-negative GC (Fig. 1D). Kaplan–Meier estimation and multivariate Cox proportional hazard model analysis showed that patients with high LINC00924 levels had shorter overall survival (OS) and progression-free survival (PFS) than patients with low LINC00924 levels (Fig. 1E, F). Moreover, LINC00924 expression was significantly correlated with lymph node status and TNM stage (Table 1). Consistent findings were obtained from the TCGA cohort (Fig. 1G–J), strengthening the validity of the results. Collectively, these results demonstrate that LINC00924 acts as an oncogene that promotes GC invasion and metastasis.

**Table 1:** the correlation of LINC00924 expression with the clinicopathological parameters of GC patients (*n* = 60).

Characteristics	Low expression LINC00924	High expression LINC00924	*p* value
	N (%)	N (%)	
Total cases	30 (100%)	30 (100%)	
Gender			
Women	10 (33%)	8 (27%)	
Man	20 (67%)	22 (73%)	0.573
Age (years)			
≥60	21 (70%)	18 (60%)	
<60	9 (30%)	12 (40%)	0.417
T-stage			
T1-T2	11 (37%)	12 (40%)	
T3-T4	19 (63%)	18 (60%)	0.791
LNM			
N0-N1	17 (57%)	1 (3%)	
N2-N3	13 (43%)	29 (97%)	*0.000*
Distant metastasis			
Yes	2 (7%)	5 (17%)	
No	28 (93%)	25 (83%)	0.228
Tumor grade			
Well, /moderate	6 (20%)	4 (13%)	
Poor	24 (80%)	26 (87%)	0.488
TNM stage			
I /II	16 (53%)	4 (13%)	
III/IV	14 (47%)	27 (87%)	*0.001*

Tumor Staging Guidelines: AJCC Cancer Staging Manual (8th). *P* < 0.05: statistically significant.

*TNM* tumor-node-metastasis, *T-Stage* tumor invasion stage.

Italic values represent *p* values < 0.05.

### LINC00924 expression promotes FAO and FA uptake

We next explored the role of LINC00924 by altering its expression in GC cells. Two shLINC00924 sequences (shLINC00924 #1 and #2) (Supplementary Table 1) were designed, and shLINC00924 #2 exhibited greater efficacy in AGS cells than shLINC00924 #1 (Supplementary Fig. 1A, B). Therefore, shLINC00924 #2 was used for subsequent experiments. LINC00924 was knocked down in AGS cells and overexpressed in MGC803 cells using lentivirus systems (Supplementary Fig. 1C). Overexpression of LINC00924 significantly increased cell proliferation, colony formation, migration, and invasion, while silencing LINC00924 had the opposite effects (Supplementary Fig. 1D–H). Collectively, these results demonstrate that LINC00924 acts as an oncogene that promotes GC invasion and metastasis.

**Fig. 2:**
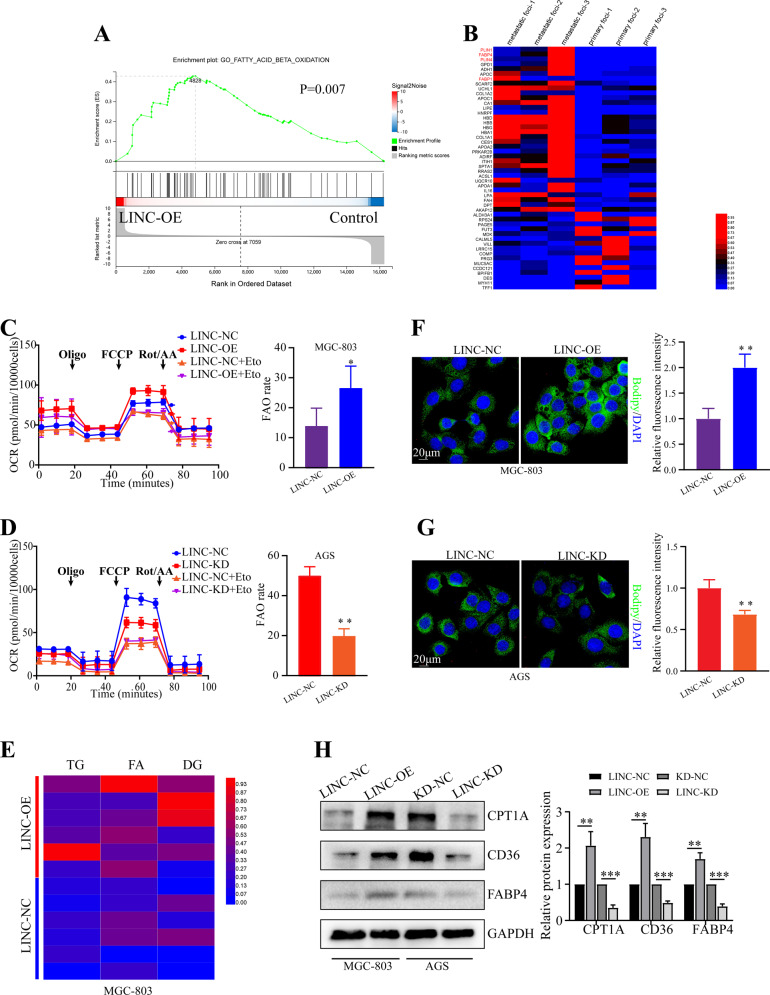
LINC00924 expression promotes FAO and FA uptake. **A** GSEA of the RNA-sequencing (RNA-seq) data from LINC00924-OE and MGC803-NC cells. **B** Differential expression of proteins in 3 pairs of GC primary lesions and peritoneal metastatic lesions determined by protein profile analysis. **C**, **D** Oxygen consumption rate (OCR) analysis in MGC803-NC/LINC-OE and AGS-NC/LINC-KD cell lines using palmitate as a metabolic substrate. MGC803 and AGS cells were treated with the CPT1 inhibitor (+ETO) or vehicle (−ETO). **E** Heatmap presenting differences in TG, FA, and DG from lipidomics between MGC803-NC and LINC-OE cell lines. **F** Lipid droplet staining in AGS-NC and LINC-OE cells. **G** Lipid droplet staining in MGC803-NC and LINC-KD cells. **H** WB analysis of CPT1A, CD36, and FABP4 in MGC803-NC and LINC-OE cells lines and in AGS-NC and LINC-KD cell lines. The protein levels were quantified with ImageJ.

To elucidate the mechanism underlying the role of LINC00924 in GC PM, we examined the biological importance of LINC00924 upregulation in GC cells. GSEA of the RNA sequencing (RNA-seq) data of MGC803-NC/LINC00924-OE cells revealed that LINC00924 overexpression upregulated numerous genes that were enriched in key pathways associated with lipid metabolism, including FA beta oxidation, FA catabolic processes and the regulation of phospholipid metabolic processes (Fig. 2A and Supplementary Fig. 2A, B). The enrichment analysis suggested that LINC00924 may be involved in lipid metabolic remodeling. Previous studies have indicated that tumor metastasis requires tumor cells to undergo a metabolic shift toward FAO [20]. In addition, our protein profile results of 3 GC patients indicated that lipid metabolism genes (such as PLIN1, PLIN4, FABP1 and FABP4) were significantly highly expressed in PM lesions compared with primary foci (Fig. 2B). Therefore, we investigated whether LINC00924 promotes GC cell metastasis through lipid metabolic reprogramming. First, we analyzed the regulation of FAO by LINC00924 using a Seahorse XFp cellular flux analyzer. We found that LINC00924 overexpression significantly increased FAO, while LINC00924 knockdown significantly decreased FAO (Fig. 2C, D). We then used targeted lipidomics to examine the effects of LINC00924 overexpression on the lipid profiles of GC cells (Supplementary Fig. 2C). LINC00924 overexpression decreased lipid species such as triglycerides (TGs), FAs and diglycerides (DGs), which participate in energy storage and metabolism (Fig. 2E). Consistent with the targeted lipidomics results, increased levels of lipid droplets were observed in the LINC00924-OE group (Fig. 2F, G). Furthermore, using TCGA data, we found a significant association between LINC00924 expression and key genes in FAO and fatty acid transport (CPT1A, CD36 and FABP4) in GC (Supplementary Fig. 3A–C). Consistent with these findings, LINC00924 upregulated CPT1A, CD36 and FABP4 (Fig. 2H). Collectively, these results indicate that LINC00924 promotes FAO and FA uptake.

### p38/PPARα signaling pathway is central to LINC00924 mediated FAO and FA uptake

**Fig. 3:**
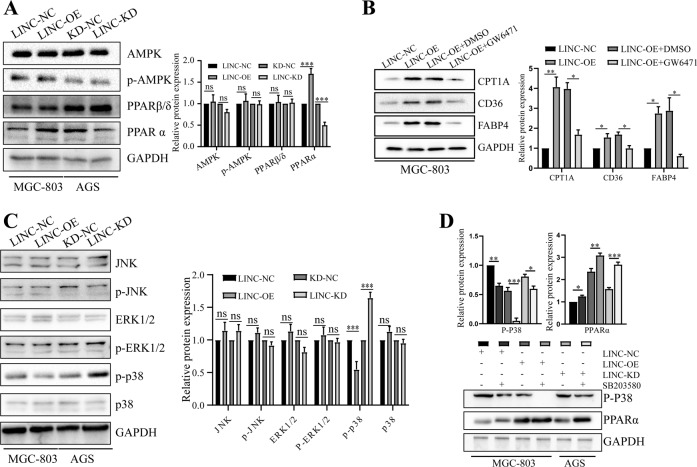
p38/PPARα signaling pathway is central to LINC00924 mediated FAO and FA uptake. **A** WB analysis of AMPK, p-AMPK, PPARα and PPARβ/δ in MGC803-NC and LINC-OE cell lines and in AGS-NC and LINC-KD cell lines. **B** WB analysis of CPT1A, CD36, and FABP4 in MGC803-NC, LINC-OE, and LINC-KD cells treated with GW6471 or DMSO. **C** WB analysis of JNK, p-JNK, ERK1/2, p-ERK1/2, p-p38, and p38 in MGC803-NC and LINC-OE cell lines and in AGS-NC and LINC-KD cell lines. **D** WB analysis of p-P38 and PPARα in MGC803-NC, LINC-OE, and LINC-KD cells treated or not treated with SB203580.

Peroxisome proliferator-activated receptors (PPARs) and AMP-activated protein kinases (AMPKs) play critical roles in cancer cell lipid metabolic reprogramming [21, 22]. Therefore, we examined the mRNA and protein levels of AMPK, PPARα and PPARβ/δ in MGC803-NC/LINC00924-OE and AGS-NC/LINC00924-KD cells. Among these proteins, only PPARα exhibited significant differences in mRNA and protein levels (Fig. 3A and Supplementary Fig. 3D). As a transcription factor, PPARα can regulate key lipid metabolic genes (including CPT1A, CD36, and FABP4) at the transcription level [23]. To determine whether LINC00924 promotes CPT1A, CD36, and FABP4 expression by regulating the expression of PPARα, we treated MGC803-NC/LINC00924 cells with the PPARα inhibitor GW6471 (10 μM, MCE, China) or DMSO for 24 h. Notably, the inhibition of PPARα by GW6471 counteracted the promotive effect of LINC00924 on CPT1A, CD36 and FABP4 expression, which is essential FAO and FA uptake for GC cells (Fig. 3B). These datas indicated that LINC00924 can activate PPARα and thus promote FAO and FA uptake.

We next investigated which signaling pathway might contribute to LINC00924-induced PPARα activation. Previous studies outlined a regulatory role for the JNK and ERK and p38 MAPK signaling pathways in regulating PPARα expression [23–26]. Therefore, we examined protein levels of the JNK, ERK and phosphorylated p38 (p-p38) MAPK in MGC803-NC/LINC00924-OE and AGS-NC/LINC00924-KD cells. We found that overexpression of LINC00924 resulted in marked suppression of p-p38 but not JNK and ERK MAPK (Fig. 3C). In contrast, knockdown of LINC00924 increased p-p38 expression (Fig. 3C). Furthermore, to test whether LINC00924 promotes PPARα expression through p38 MAPK, we treated LINC00924-OE, LINC00924-KD, and control cells with the specific p38 MAPK inhibitor SB203580. The results demonstrated that SB203580 (10 μM, MCE, China) clearly increased PPARα expression, especially in LINC00924-KD cells (Fig. 3D). Taken together, these results demonstrate that LINC00924 facilitates lipid metabolic reprogramming through the p38 MAPK/PPARα pathway.

### LINC00924 physically interacts with hnRNPC

**Fig. 4:**
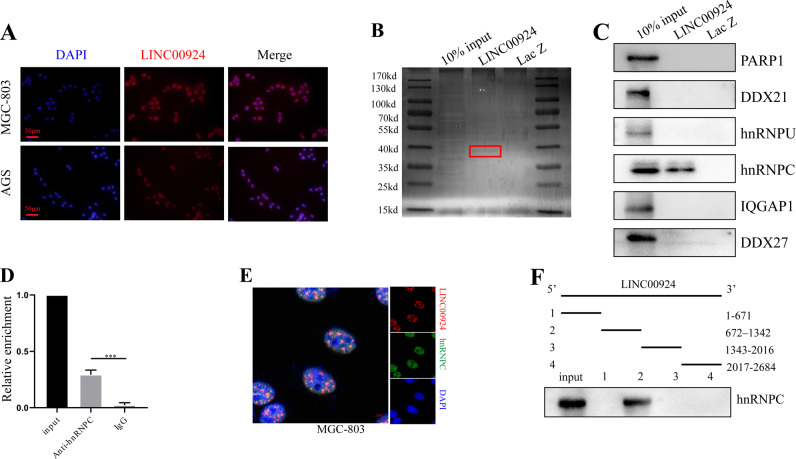
LINC00924 physically interacts with hnRNPC. **A** FISH results showing the nuclear localization of LINC00924 (red) in MGC803 and AGS cells. The nuclei were stained with DAPI. **B** Silver staining of biotinylated LINC00924-interacting proteins. Red boxes indicate specific bands. **C** WB results used to confirm the mass spectrometry results. **D** RIP analysis conducted using an anti-hnRNPC antibody to validate the interaction between hnRNPC and LINC00924. **E** Confocal RNA-FISH (red) and immunofluorescence (IF) (green) images indicating the colocalization of LINC00924 and hnRNPC. **F** Results of the LINC00924 serial deletion assay performed to localize the interaction region.

We next sought to explore the underlying molecular mechanisms by which LINC00924 promotes FAO and FA uptake. Nuclear/cytoplasmic RNA isolation and FISH revealed that LINC00924 was mainly localized in the nucleus (Fig. 4A and Supplementary Fig. 3E). Nuclear lncRNAs can exert their activities by interacting with RNA-binding proteins [14, 27]. Thus, we performed RNA pulldown assays and mass spectrometry to identify potential interacting proteins (Fig. 4B). A total of 269 potential interacting proteins were identified (Supplementary data 2), and the 6 top-ranked proteins were selected for WB validation. Only hnRNPC interacted with LINC00924 (Fig. 4C). First, hnRNPC was detected in samples of adjacent normal tissue (ANT), primary GC, and PM (*n* = 3) by WB. The results showed that hnRNPC protein were significantly upregulated in PM compared to that in GC and ANT (Supplementary Fig. 3F).

To verify the physical interaction between LINC00924 and hnRNPC, we performed a RIP assay with an anti-hnRNPC antibody. The qRT–PCR results showed that LINC00924 was significantly enriched in hnRNPC immunoprecipitates but not in IgG immunoprecipitates (Fig. 4D). Furthermore, confocal RNA-FISH and immunofluorescence images showed the colocalization of LINC00924 and hnRNPC (Fig. 4E). Finally, a LINC00924 serial deletion assay was performed to localize the interaction region. The RNA pulldown results showed that the LINC00924 fragment 672–1342 but not fragments 1–671, 1343–2016, or 2017–2684 coprecipitated with hnRNPC (Fig. 4F). These experimental results revealed that hnRNPC is a LINC00924-associated protein. However, LINC00924 did not modify hnRNPC levels or stability (Supplementary Fig. 3G, H). Previous studies [28, 29] showed that hnRNPC could regulate cancer-specific alternative cleavage, which suggested that LINC00924 might regulate premRNA splicing via hnRNPC.

### LINC00924 regulates the P38/PPARα signaling pathway via Mnk2 pre-mRNA AS

**Fig. 5:**
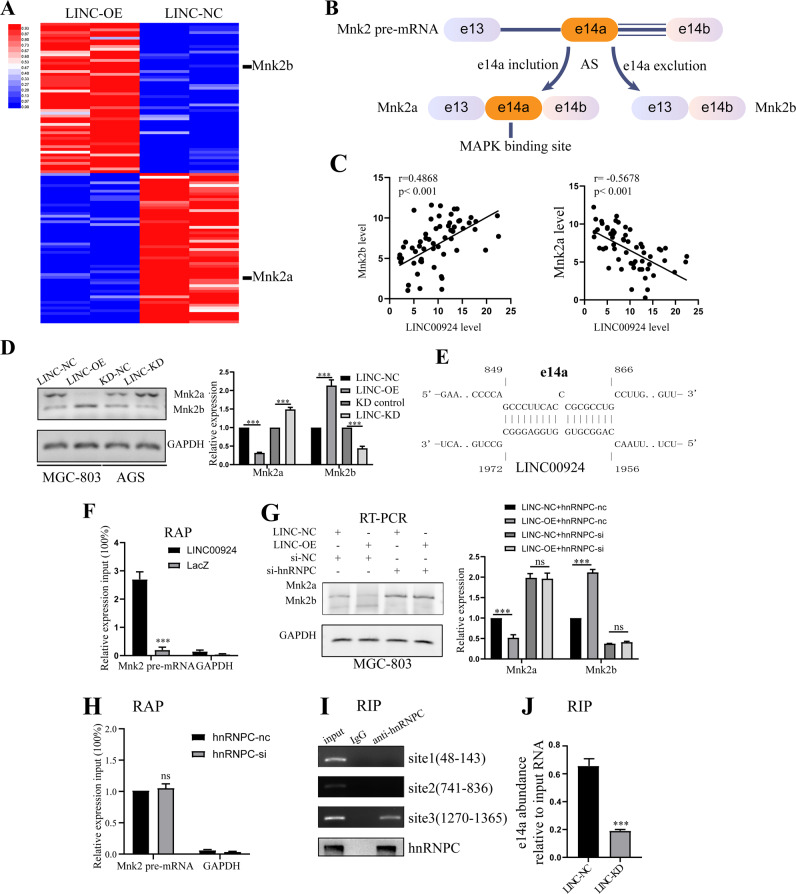
LINC00924 regulates the P38/PPARα signaling pathway via hnRNPC regulated Mnk2 pre-mRNA AS. **A** Heatmap presenting significantly differentially expressed transcripts between MGC803-NC and LINC00924-OE cell lines. **B** A schematic model of Mnk2 AS. MNK2 pre-mRNA can be alternatively spliced to yield two isoforms: Mnk2a and Mnk2b. Mnk2a contains e14a, a binding site for MAP kinases, while Mnk2b does not [30]. **C** The correlations of LINC00924 expression with Mnk2b (**B**) and Mnk2a (**C**) expression in GC tissues. **D** RT–PCR analysis of Mnk2a and Mnk2b in MGC803-NC and LINC-OE cell lines and in AGS-NC and LINC-KD cell lines. **E** Schematic illustration of base pairing between LINC00924 and the e14a of Mnk2. **F** qRT–PCR detection of e14a enrichment by LINC00924 using a RAP assay. **G** RT–PCR analysis of MGC803-NC and LINC-OE cell lines treated with si-hnRNPC or NC. **H** hnRNPC knockdown did not disrupt the RNA–RNA duplex formed by LINC00924 and e14a. **I** RIP analysis conducted using an anti-hnRNPC antibody to validate the interaction between hnRNPC and the e14a of Mnk2. **J** LINC00924 is essential for the formation of the RNA–RNA duplex by LINC00924 and e14a.

To explore the molecular mechanisms by which LINC00924 regulates P38/PPARα, RNA transcriptome sequencing was performed in LINC00924-OE and control MGC803 cells. We identified 115 differentially expressed transcripts (i.e., transcripts having a greater than 2-fold difference in expression), including 50 downregulated transcripts and upregulated 65 transcripts in LINC00924-OE cells relative to control cells (Supplementary data 1). Among these differentially expressed transcripts, Mnk2a and Mnk2b, which were downregulated and upregulated, respectively, in LINC00924-OE cells, attracted our attention (Fig. 5A). As previously reported [30–32], Mnk2 pre-mRNA can be alternatively spliced to yield two isoforms: Mnk2a and Mnk2b. Mnk2a contains a MAPK-binding site, whereas Mnk2b does not (Fig. 5B) [33]. Moreover, Mnk2 pre-mRNA AS is a well-known regulator of p38 MAPK activation [30, 31]. Therefore, we next investigated whether LINC00924 regulates the P38/PPARα signaling pathway via Mnk2 pre-mRNA AS. First, we examined Mnk2a and Mnk2b expression in GC samples. The results showed there is a significant positive/negative correlation between LINC00924 and Mnk2b/Mnk2a expression (Fig. 5C). Consistent with our RNA-seq results, LINC00924 overexpression significantly increased Mnk2b expression and substantially attenuated Mnk2a expression (Fig. 5D). Moreover, using the IntaRNA program (http://rna.informatik.uni-freiburg.de), we identified a LINC00924 potential binding site at e14a of Mnk2 pre-mRNA (Fig. 5E). RAP experiments were performed to verify the physical interaction between LINC00924 and e14a of Mnk2 pre-mRNA, and the results showed e14a enrichment in LINC00924 group (Fig. 5F). Taken together, these results revealed that LINC00924 can regulate Mnk2 pre-mRNA AS.

### LINC00924 regulates Mnk2 pre-mRNA AS via hnRNPC

We next sought to explore whether LINC00924 regulates Mnk2 pre-mRNA AS via hnRNPC. Collectively, our results showed that LINC00924-induced Mnk2 pre-mRNA AS was partially blocked by hnRNPC knockdown (Fig. 5G and Supplementary Fig. 3I). These results suggested that LINC00924 might regulate Mnk2 pre-mRNA splicing via hnRNPC.

We next wanted to investigate the mechanism by which LINC00924/hnRNPC regulates Mnk2 pre-mRNA AS. First, we found that hnRNPC knockdown did not disrupt the RNA–RNA duplex formed by LINC00924 and the e14a of Mnk2 pre-mRNA (Fig. 5H). Previous studies have indicated that hnRNP proteins can bind to intronic regions to enhance or repress splicing [34]. Here, we identified three hnRNPC-binding sites in Mnk2 pre-mRNA within e14a using the catRAPID algorithm. RIP assays verified the interaction of hnRNPC and the e14a of Mnk2 pre-mRNA at, site3, but not site 1 or 2 (Fig. 5I). Next, we explored whether this interaction was dependent on LINC00924. Interestingly, the interaction of hnRNPC and the e14a of Mnk2 was significantly attenuated by LINC00924 knockdown (Fig. 5J). In summary, our results indicate that LINC00924 can promote hnRNPC binding to Mnk2 pre-mRNA at e14a, thus downregulating Mnk2a splicing.

### Mnk2 AS is essential for LINC00924 regulation of lipid metabolic reprogramming

**Fig. 6:**
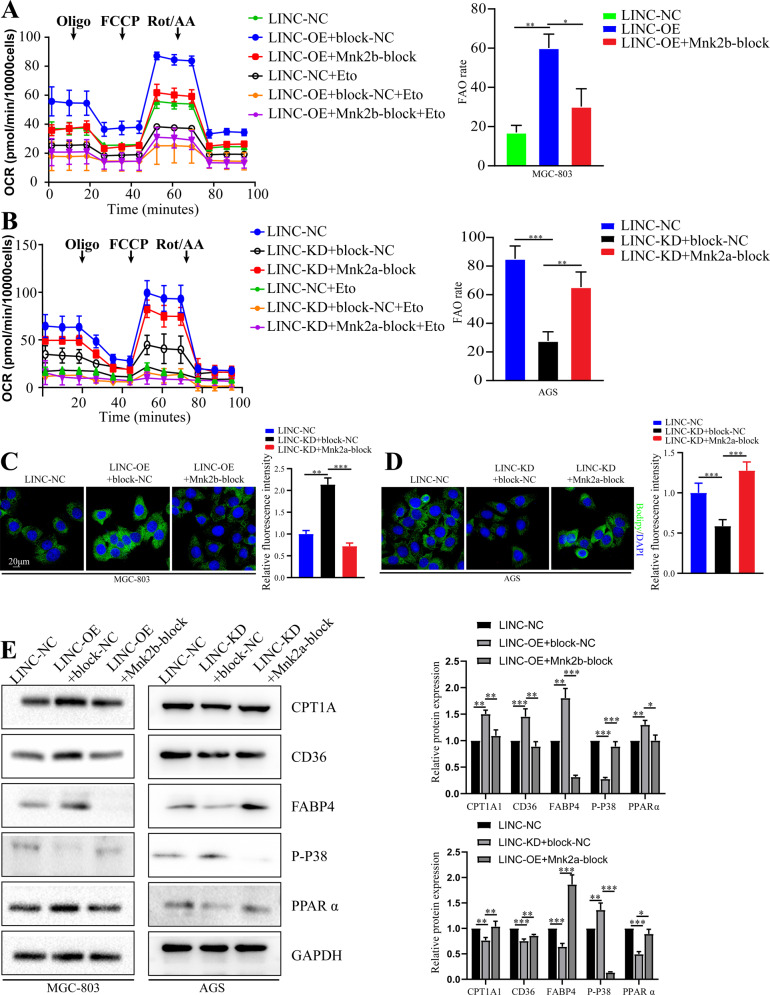
Mnk2 AS is essential for LINC00924 regulating lipid metabolic reprogramming. **A** OCR analysis of MGC803-NC and LINC-OE cell lines treated with Mnk2b-block SSO or NC using palmitate as a metabolic substrate. **B** OCR analysis of AGS-NC and LINC-KD cell lines treated with Mnk2a-block SSO or NC using palmitate as a metabolic substrate. **C** Lipid droplet staining of AGS-NC and LINC-OE cells treated with Mnk2a-block SSO or NC. **D** Lipid droplet staining if MGC803-NC and LINC-KD cells treated with Mnk2a-block SSO or NC. **E** WB analysis of CPT1A, CD36, FABP4, p-P38, and PPARα in MGC803-NC and LINC-OE cells treated with Mnk2b-block SSO or NC and AGS-NC and LINC00924-KD cells treated with Mnk2a-block SSO or NC.

We next explored whether Mnk2 AS plays a critical role in LINC00924-induced lipid metabolic reprogramming. To modulate MNK2 splicing, 2′-OMe SSOs, which mask the 3′ splice sites of alternative exons 14a and 14b of MNK2, were used to shift the ratio of Mnk2a and Mnk2b mRNA isoforms (Supplementary Fig. 4A) [31]. The 2a-block SSO inhibited the Mnk2a isoform, and the 2b-block SSO upregulated the Mnk2a isoform (Supplementary Fig. 4B). As shown, LINC00924 overexpression facilitated FA transport and FAO, and the function of LINC00924 was abrogated by Mnk2b-block. In contrast, Mnk2a-blockade also rescued LINC00924-induced lipid metabolic reprogramming (Fig. 6A–D). These results were validated by the WB analysis of CPT1A, CD36 and FABP4 (Fig. 6E).

### LINC00924 promotes matrix-detached GC cell survival and metastasis in vitro and in vivo

**Fig. 7:**
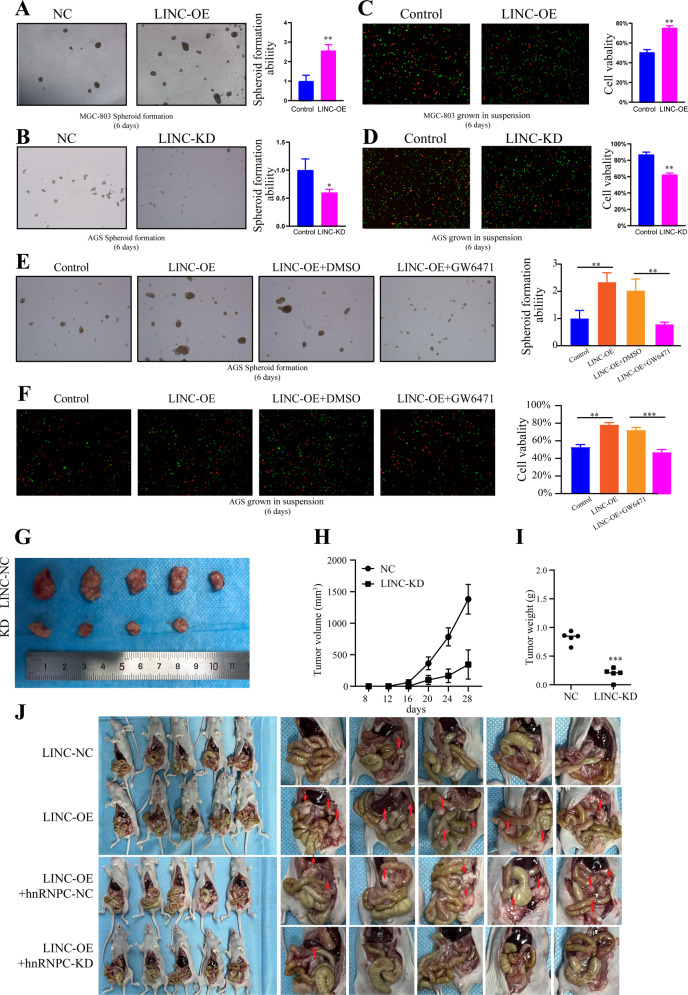
LINC00924 promotes matrix-detached GC cells survival and metastasis in vitro and in vivo. **A** 3D spheroid formation assay of MGC803-NC and LINC-OE cells. Results were quantified as fold changes. **B** 3D spheroid formation assay of AGS-NC and LINC-KD cells. Results were quantified as fold changes. **C** Live and dead staining of MGC803-NC and LINC-OE cells after culture on 3D ultralow attachment plates for 6 days (with live cells stained in green and dead cells stained in red). Results were quantified as fold changes. **D** Live and dead staining of AGS-NC and LINC-KD cells after culture on 3D ultralow attachment plates for 6 days (with live cells stained in green and dead cells stained in red). Results were quantified as fold changes. **E** 3D spheroid formation assay of MGC803-NC and LINC-OE cells treated or not treated with GW6471. Results were quantified as fold changes. **F** Live and dead staining of AGS-NC and LINC-KD cells treated or not treated with GW6471 after culture on 3D ultralow attachment plates for 6 days (with live cells stained in green and dead cells stained in red). Results were quantified as fold changes. **G** The morphological characteristics of subcutaneous tumor xenografts in the AGS-NC and LINC-KD groups. **H** Subcutaneous tumor volume in the AGS-NC and LINC-KD groups. **I** Subcutaneous tumor weight in the AGS-NC and LINC-KD groups. **J** The morphological characteristics of peritoneal tumor xenografts in the LINC-NC, LINC-OE, LINC-OE + hnRNPC-NC and LINC-KD + hnRNPC-KD groups.

GC PM is the process in which GC cells shed from the primary tumor, survive as latent tumor-initiating seeds, and eventually break out to replace the host tissue [6, 20]. The survival of matrix-detached GC cells is a prerequisite for PM. To mimic the in vivo microenvironment during GC cell detachment from primary lesions, cells were cultured on 3D ultralow attachment plates for 6 days. Cell viability and spheroid formation were assessed. LINC00924 overexpression significantly promoted matrix-detached GC cell survival and spheroid formation. In contrast, LINC00924 knockdown led to significant decrease in cell survival and spheroid formation (Fig. 7A–D). Subsequently, to confirm that lipid metabolic reprogramming is essential for LINC00924-induced matrix-detached GC cell survival and spheroid formation, we used the PPARα inhibitor GW6471, which suppresses FAO and FA uptake. As expected, LINC00924-induced matrix-detached GC cell survival, and spheroid formation was reversed by GW6471 (Fig. 7E, F).

To validate the role of LINC00924 in tumor growth in vivo, a nude mouse xenograft tumor model was constructed. LINC-NC AGS cells and LINC-KD AGS cells were injected subcutaneously into nude mice. The results revealed that knockdown of LINC00924 markedly prevented subcutaneous tumor growth (Fig. 7G–I). To further prove that LINC00924 facilitates gastric cancer peritoneal metastasis via hnRNPC, we constructed stable LINC-NC, LINC-OE, LINC00924-OE + hnRNPC-NC and LINC00924-KD + hnRNPC-KD in MGC-803 cells (5 × 106 cells in 40 μl of serum-free medium containing 50% Matrigel), which were subserosal injected into the greater curvature of the stomach (Supplementary Fig. 4C). After 35 days, the mice were sacrificed, and the expression of CPT1A, CD36, FABP4, PPARα, p-p38, hnRNPC, Mnk2a, and Mnk2b, in peritoneal xenograft tumors was analyzed. Result showed that LINC00924 overexpression effectively promoted intraperitoneal tumor dissemination, and hnRNPC knockdown rescued LINC00924-induced intraperitoneal tumor dissemination (Fig. 7J). IHC staining analysis, WB analysis and RT-PCT analysis of intraperitoneal disseminated tumors revealed overexpressed expression of CPT1A, CD36, FABP4, PPARα and Mnk2b, and decreased expression of p-p38 and Mnk2b expression in the LINC-OE group, which was rescued by hnRNPC knockdown (Supplementary Fig. 4D, E). Taken together, these results proved that LINC00924 facilitates gastric cancer peritoneal metastasis via hnRNPC-regulated alternative splicing of Mnk2 in vivo.

## Discussion

PM is a main cause of mortality in patients with GC, and no effective prevention or therapeutic strategies are currently available in the clinic. Elucidating the mechanism of GC PM could be beneficial for prevention and treatment of GC patients. Over the past decade, an increasing number of lncRNAs have been shown to drive cancer metastasis, such as lncRNA HOXA11-AS and LINC01234 [27, 35]. In this study, we demonstrated that LINC00924 was more highly expressed in PM-positive GC patients than in PM-negative GC patients, and that its high expression mediates a broad range of PMs. LINC00924 regulated GC cell lipid metabolic reprogramming, which subsequently promoted matrix-detached GC cell survival and spheroid formation. Regarding the mechanism, LINC00924 regulated MNK2 pre-mRNA AS by binding to hnRNPC, thus regulating the p38 MAPK/PPARα signaling pathway. Specifically, LINC00924 promoted hnRNPC binding to MNK2 pre-mRNA at e14a, which downregulated Mnk2a splicing. Finally, we demonstrated that LINC00924 was essential for GC cell growth and PM formation and colonization in vivo. Our results extend the understanding of lncRNA-mediated cancer metastasis and may aid the prevention and treatment of GC PM.

Previously, we have identified LINC00924 as a GC PM-related lncRNA through lncRNA expression profiles in GC primary foci and peritoneal foci. In this article, we further proved that LINC00924 could play an oncogenic role in gastric cancer. However, a recent publication suggested that LINC00924 functioned as tumor suppressor via sponging miR-6755–5p in hepatitis B virus-related hepatocellular carcinoma [36]. Our study shows that LINC00924 was mainly located in the nucleus. The conflicting results regarding the role of LINC00924 in human malignancies may be attributed to the diverse properties of different cancers and stages of tumor progression [27]. Recently, certain lncRNAs have been shown to play important roles in the regulation of AS during tumor invasion and metastasis. For example, PLANE regulates NCOR2 AS by forming an RNA–RNA duplex with NCOR2 pre-mRNA to promote cancer pathogenesis [37]; LINC01348 suppresses hepatocellular carcinoma metastasis by inhibiting SF3B3-mediated EZH2 pre-mRNA splicing [38], and DGCR5 promotes oncogenesis in esophageal squamous cell carcinoma via SRSF1-mediated AS of Mcl-1 [39]. Splice factors are the crux of lncRNA-induced pre-mRNA AS [14]. Likewise, our results demonstrated that LINC00924 regulates Mnk2 AS by binding to the splice factor hnRNPC. We found that LINC00924 formed an RNA–RNA duplex with the Mnk2 pre-mRNA within e14a, which promoted hnRNPC binding to Mnk2 pre-mRNA at e14a and downregulated Mnk2a splicing. These results provide insight into the mechanism of lncRNA-mediated gene regulation.

Mnk2 has been reported to be involved in the pathogenesis of various cancers [31, 32, 40], but its function in GC has not been investigated. The splice factor SRSF1 has been shown to promotes Mnk2 splicing into Mnk2b instead of Mnk2a, and thereby enhance tumor proliferation in colon adenocarcinoma [32]. Consistent with these findings, we found that Mnk2a splicing was downregulated in GC and regulated by the LINC00924/hnRNPC axis. However, distinct from previous studies that focused on cell growth and oncogenesis, this study revealed that the downregulation of Mnk2a splicing promotes FAO and FA uptake, which promote GC PM. This finding extends our understanding of the function of Mnk2 during the development of oncogenesis, and identifies Mnk2 as a potential therapeutic target for GC PM treatment.

GC PM is a multistep process, it includes the detachment of cells from the primary tumor, peritoneal transport, mesothelial adhesion, invasion of the submesothelial tissue and systemic metastasis. The survival and tumor-initiating activity of metastatic cells are critical in driving or supporting PM [41]. Recently, several studies have demonstrated that lipid metabolic remodeling facilitates metastatic cell growth and survival. For example, Ser et al. found that FAO was a druggable factor that regulates cellular plasticity to drive metastasis in breast cancer [42], and Li et al. reported that the nuclear receptor Nur77 facilitates melanoma cell survival under metabolic stress by protecting FAO [43]. Here, we found that LINC00924 can moderate lipid metabolic remodeling, with effects including increased FA uptake and FAO, which are essential for matrix-detached GC cell survival and spheroid formation.

There are several limitations in the current study. First, we focused on lipid metabolic reprogramming of LINC00924; however, it is possible that LINC00924 might also regulate other biological functions in GC PM. Second, the underlying mechanism by which metastatic cells maintain high LINC00924 expression remains incompletely understood. Finally, it remains unclear whether LINC00924-induced lipid metabolic reprogramming is GC-specific or it also present in other types of cancer; more studies are needed to clarify this issue.

## Conclusion

**Fig. 8:**
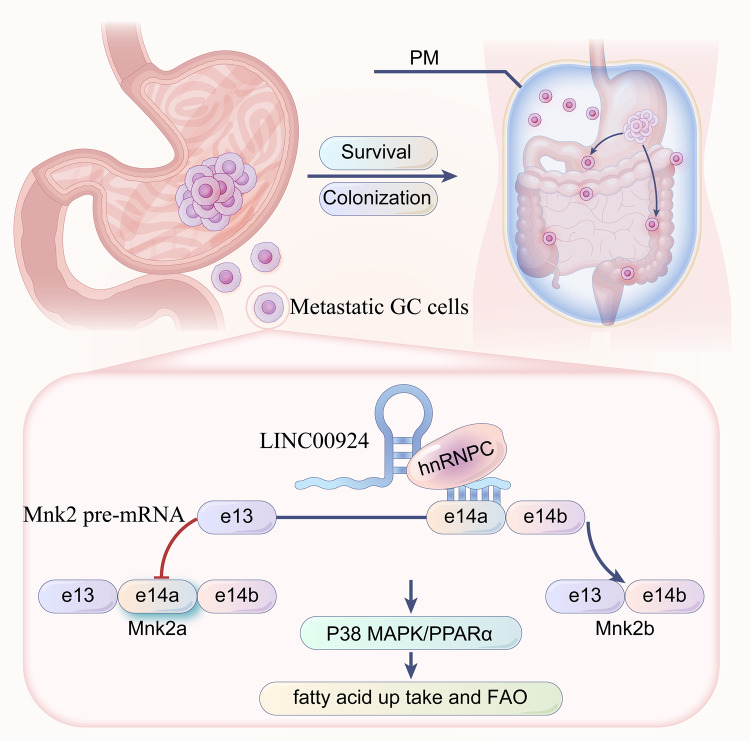
LINC00924-induced fatty acid metabolic reprogramming facilitates gastric cancer peritoneal metastasis via hnRNPC-regulated alternative splicing of Mnk2.

In summary, we identified LINC00924 as a GC PM-related lncRNA. We found that LINC00924 regulated GC cell lipid metabolic reprogramming, which subsequently promoted matrix-detached GC cell survival and spheroid formation. We found that LINC00924 was essential for GC cell growth and PM formation in vitro and in vivo. Our results extend the understanding of lncRNA-mediated cancer metastasis and may be potentially efficacious for the prevention and treatment of GC PM (Fig. 8).

## Supplementary information

Supplemental Supplemental

Supplemental data1

Supplemental data 2

AJ Cheklist

## Data Availability

The original RNA-seq data of MGC803-NC/LINC00924-OE have been deposited in the database of the NCBI Sequence Read Archive (https://www.ncbi.nlm.nih.gov/sra/) under the accession number PRJNA819555.
