# Supplementary material for: LINC00924-induced fatty acid metabolic reprogramming facilitates gastric cancer peritoneal metastasis via hnRNPC-regulated alternative splicing of Mnk2
Source: Cell Death Dis. 2022 Nov 23;13(11):987. doi: 10.1038/s41419-022-05436-x (PMC9684446; doi:10.1038/s41419-022-05436-x)
Supplement: Supplementary file 1 — Supplemental Supplemental [file 41419_2022_5436_MOESM1_ESM.docx]

**LINC00924-induced fatty acid metabolic reprogramming facilitates gastric cancer peritoneal metastasis via hnRNPC-regulated alternative splicing of Mnk2**

Qiuming He^1,2,3,4†^, Chaogang Yang^1,2,3,4†^, Zhenxian Xiang^1,2,3,4†^, Guoquan Huang^1,2,3,4^, Haitao Wu^1,2,3,4^, Tingna Chen^1,2,3,4^, Rongzhang Dou^1,2,3,4^, Jialing Song^1,2,3,4^, Lei Han^1,2,3,4^, TianTian Song^1,2,3,4^, Shuyi Wang^1,2,3,4*^ and Bin Xiong ^1,2,3,4*^

1 Department of Gastrointestinal Surgery, Zhongnan Hospital of Wuhan University, No.169 Donghu Road, Wuchang District, Wuhan, 430071, China.

2 Department of Gastric and Colorectal Surgical Oncology, Zhongnan Hospital of Wuhan University, No.169 Donghu Road, Wuchang District, Wuhan, 430071, China.

3 Hubei Key Laboratory of Tumor Biological Behaviors, No.169 Donghu Road, Wuchang District, Wuhan, 430071, China.

4 Hubei Cancer Clinical Study Center, No.169 Donghu Road, Wuchang District, Wuhan, 430071, China.

† Qiuming He, Chaogang Yang and Zhenxian Xiang contributed equally to this work.

* Correspondence: Bin Xiong, [binxiong1961@whu.edu.cn](mailto:binxiong1961@whu.edu.cn).

* Correspondence: Shuyi Wang, [shuyiwang@whu.edu.cn](mailto:shuyiwang@whu.edu.cn).

**Supplementary Figure 1**

A: qRT-PCR analysis of LINC00924 expression level in GES-1 and GC cells lines.

B-C: Construction of the LINC00924-KD/OE stable cell lines.

D: Colony formation assays were performed after LINC00924 knockdown or overexpression in AGS or MGC-803, respectively.

E-F: Migration and invasion assays of GC cell lines after LINC00924 knockdown or overexpression.

G: Wound healing assay was performed in GC cell lines following LINC00924 knockdown or overexpression.

H: CCK8 assay was performed in GC cell lines following LINC00924 knockdown or overexpression

**Supplementary Figure 2**

A-B: GSEA analyses of the RNA-sequencing (RNA-seq) data from LINC00924 OE and NC MGC803 cells.

C: Heatmap represents all differential expressed lipid metabolites among MGC803-NC/LINC-OE cell lines.

**Supplementary Figure 3**

A-C: The correlation of LINC00924 expression with CPT1A, CD36 and FABP4 expressions in GC tissues from TCGA.

D: qRT-PCR analysis of AMPK, PPARα and PPARβ/δ in MGC803-NC/LINC-OE and AGS-NC/ LINC-KD cell lines.

E: Subcellular fractionation analyses of LINC00924 in MGC803 and AGS cell lines.

F: hnRNPC protein expressions in 3 pairs GC peritoneal metastasis samples.

G: LINC00924 does not affect the colocalization of hnRNPC.

H: LINC00924 does not affect the expression of hnRNPC.

I: Efficiency of hnRNPC knockdown and si-3 was applied for subsequent experiments.

**Supplementary Figure 4**

A: Schematic diagram of splice site-interfering RNA oligonucleotides.

B: Efficiency of Mnk2a-block and Mnk2b-block.

C: Tumor peritoneal dissemination assay in nude mice.

D: Immunohistochemistry of PPARα, p-p38, CPT1A, CD36, FABP4 and hnRNPC in peritoneal tumor xenografts.

E: WB or RT-PCR analysis of PPARα, p-p38, CPT1A, CD36, FABP4, hnRNPC, Mnk2a and Mnk2b in peritoneal tumor xenografts.

**Supplementary Tables**

**Supplementary Table 1.** **List of siRNAs/shRNAs**

| LINC00924 | shRNA.1: GACATCTCATTTGTTTTAACTAT  shRNA.2: GGAGACAATACGGCCAATTAA |
| --- | --- |
| hnRNPC | siRNA.1: GCCTTCGTTCAGTATGTTAAT  siRNA.2: GCGCTTGTCTAAGATCAAATT  siRNA.3: CTCCTGTATATTTTAAATCATCT |

**Supplementary Table 2. Antisense RNA oligos (all bases 2’-O-methyl):**

| Mnk2a block oligo | CAGCTGTTCCTGGGAAACGGGG |
| --- | --- |
| Mnk2b block oligo | GGAAGTGACTGTCCCACCTTCAGA |

**Supplementary Table 3. List of antibodies**

| Antibody | Catalogue No. | company |
| --- | --- | --- |
| FABP4 | 12802-1-AP | PROTEINTECH |
| CD36 | 18836-1-AP | PROTEINTECH |
| JNK | 66210-1-Ig | PROTEINTECH |
| p-JNK | 80024-1-RR | PROTEINTECH |
| AMPK | 66536-1-Ig | PROTEINTECH |
| CPT1A | 15184-1-AP | PROTEINTECH |
| p-p38 | 4511T | Cell Signaling Technology |
| P38 | 8690T | Cell Signaling Technology |
| p-ERK1/2 | 8544T | Cell Signaling Technology |
| ERK1/2 | 4695T | Cell Signaling Technology |
| p-AMPK | 2535T | Cell Signaling Technology |
| hnRNPC | ab133607 | ABCAM |
| PPARα | ab178866 | ABCAM |
| PPARβ/δ | ab178865 | ABCAM |

**Supplementary Table 4. List of qRT-PCR primers**

| LINC00924 | Forward: AGTGCAGACAGCCGTTAAGT  Reverse: TTAGGGTAGTGTCAGCCGGA |
| --- | --- |
| PPARα | Forward: ATGGTGGACACGGAAAGCC  Reverse: CGATGGATTGCGAAATCTCTTGG |
| PPARβ/δ | Forward: CAGGGCTGACTGCAAACGA  Reverse: CTGCCACAATGTCTCGATGTC |
| GAPDH | Forward: GGAGCGAGATCCCTCCAAAAT  Reverse: GGCTGTTGTCATACTTCTCATGG |
| Mnk2a | Forward: TCCGTGACGCCAAGCAG  Reverse: GGTCTTTGGCACAGCTG |
| Mnk2b | Forward: TCCGTGACGCCAAGCAG  Reverse: GAGGAAGTGACTGTCCCAC |
| AMPK | Forward: ACCAGGTCATCAGTACACCATC  Reverse: TCTGGTGCAGCATAGTTGGG |
| E14a of Mnk2 | Forward: GCCTCCGACCTTGACCTTAAA  Reverse: CCAAGGGGGTGTCTTCAGGA |

**Supplementary Table 5. List of RT-PCR primers**

| Mnk2a | Forward: GCTGCGACCTGTGGAGCCTGGG  Reverse: GATGGGAGGGTCAGGCGTGGTC |
| --- | --- |
| Mnk2b | Forward: GCTGCGACCTGTGGAGCCTGGG  Reverse: GAGGAGGAAGTGACTGTCCCAC |
| E14a of Mnk2 (site1) | Forward: CATTGCCATGAACCGGCAG  Reverse: GAGGTAGCTCGGACCAGGAC |
| E14a of Mnk2 (site2) | Forward: AGGCTCTTCCCCTCTATCCC  Reverse: CCTGACACCTCCAGAGACAG |
| E14a of Mnk2 (site3) | Forward: GCTCAGTCTGACCGTAGCCA  Reverse: TCTGGGCTCAGCTCTAAGGA |
